# Online flipped learning methods for teaching hospitality skills and management practices in an epidemic situation: A study on learning attitude and effectiveness

**DOI:** 10.3389/fpsyg.2022.915992

**Published:** 2023-01-18

**Authors:** Kuo-Wei Chen, Ze-Yung Wang

**Affiliations:** ^1^Department of Hospitality Management, Ming Chuan University, Taoyuan, Taiwan; ^2^Department of Hospitality Management, Tajen University, Pingtung, Taiwan

**Keywords:** flipped learning, hospitality management learning, learning effectiveness, learning attitude, cognition component

## Abstract

Starting in 2019, the ongoing COVID-19 pandemic has lasted 3 years and will likely continue to affect the lives of people all over the world. According to a United Nations Educational, Scientific and Cultural Organization (UNESCO) survey, more than 91% of students from all over the world have been affected by the spread of COVID-19. The application of technological networks can help solve problems related to being unable to attend school in person, as online teaching can effectively help reduce learning loss in the short term. In Taiwan, the higher education system has been using online learning, but now faces a new and huge crisis, as some courses do not readily translate to this setting. In professional courses run by hospitality departments, it is essentially impossible to accurately convey the practical skills required, for example, aspects of color, aroma, and taste through online teaching. Moreover, the learning level of each student varies greatly. During the online teaching process, instructors teach professional skills and movements through a single teaching video, which may not meet the needs of all students. In response, this study explores using the flipped teaching method, to not only enable students to master and control their learning and effectively adjust their self- adaptive learning progress but also to help teachers solve problems and impart professional skills using a two-way, interactive, online teaching method. This approach, flipping a class in an online learning environment, could effectively make up for the one-way teaching sometimes created by video content, and address the problem of gaps in learning professional practical skills. It can also induce students with poor learning attitudes to actively participate in learning. This study involved 55 bachelor students from a university of science and technology in Taiwan. The research results are as follows: (1) Students who participated in the flipped teaching mode, which involved two-way interaction showed better professional understanding of the course and improved willingness to learn, thereby improving the learning effect. (2) Awareness of these poor practical catering professional skills in students, assisted in laying the professional foundation for students to gradually improve their learning attitude and their advanced skills. This indicates that students with poor academic performance in an online environment might benefit from two-way interactive teaching. Teachers should clarify detailed descriptions of professional practical actions that confuse students. (3) In flipped learning, the grouping of “game/toy-based e-learning” can not only improve the performance of students who actively study to achieve good grades but also help and motivate other students to learn together. These results indicate that in flipped classrooms that use an online learning environment, the active learning and learning attitudes of students were positive and that their interest in learning and learning efficiency was also significantly improved. At the same time, this approach stimulated the innovation, creativity, and creative development of students in using professional technology in the hospitality industry. It transformed the passive learning situation of online one-way teaching into an active two-way teaching environment.

## Introduction

Countries throughout the world are actively changing the teaching model of higher education in order to effectively teach higher education students, and retain them in a fiercely competitive global higher education market (Segura-Robles et al., 2020). Through the analysis of Web of Science we found that more than 2,968 studies have focused on flipped learning and flipped classroom approaches (López-Belmonte et al., 2022). This indicates that flipped learning has attracted widespread attention around the world and is an increasingly popular method of classroom learning in university education today (Pozo-Sánchez et al., 2022). Flipped learning is a student-centered learning and teaching activity that reverses the traditional classroom situation (Singay, 2020). This blended teaching method, in terms of teaching potential and teaching effectiveness, has attracted the attention of the education and teaching sector (Zainuddin et al., 2019; López-Belmonte et al., 2022). In the process of flipped learning, through the inverted learning method, teachers can make full use of classroom time, deepen the depth and breadth of learning, and build students’ self-knowledge (El Miedany, 2019; Marín Marín et al., 2021). Coupled with the global impact of COVID-19, higher education has been subject to an unprecedented number of school closures, meaning that distance and online teaching models have mushroomed (Chukwuemeka et al., 2021; Gumbheer et al., 2022). Taiwan entered level 3 alerts due to COVID-19 in March 2020, and universities were forced to implement distance learning without warning. Even though these measures aimed to have a minimum impact and protect students’ rights to education, it has been difficult to identify learning difficulties and learning outcomes on some professional and technical courses, and both teachers and students have experienced considerable difficulties that have significantly impacted education (Ye and Ye, 2020). Flipped learning is regularly used in social and humanities, science and engineering, natural science, biomedicine, and other disciplines, and students have performed well in these contexts (Tien et al., 2020). However, the application of flipped teaching in the distance online teaching of hospitality management still needs to be studied through empirical analysis to confirm its effectiveness on learning attitude and learning effectiveness. This study included 55 undergraduate students in their third grade of the department of hospitality management of a university of science and technology in Taiwan, who experienced a total of 32 hours of flipped learning over 8 weeks. It aimed to explore whether long-distance online teaching can complement and strengthen flipped learning in hospitality management.

## Literature

### Distance learning development

In Taiwan, distance education has a long history and has shown good results, from early correspondence teaching, radio broadcast and TV broadcast courses to online distance teaching in various universities (Xie et al., 2022). Academics claim that distance teaching is more effective than face-to-face teaching, and it can replace face-to-face courses (Yilmaz, 2019). However, further investigation and confirmation are needed to determine whether distance learning is suitable for all types of courses and for all students (Hsiao, 2021). General knowledge or basic subjects, such as Chinese, history, society, and classical physical chemistry, can be viewed and learned through repeated observation and can also achieve quite good learning outcomes (Tang, 2021). However, distance teaching has rarely been offered for technical courses because of the differences in students’ learning ability and also mainly because these courses require students to practice using equipment, learning basic skills, and undertake complex tasks. If coupled with the students’ proficiency in distance teaching, it will directly affect the teaching results (Hsiao, 2021; Tang, 2021). Although the use of distance teaching methods in technical courses has proved quite effective, the learning effect is not obvious (Tang, 2021). Distance teaching has been developed and applied to general knowledge or basic subjects, but the experiences of teachers are difficult to replicate in distance teaching projects on technical courses.

### The application of flipped learning in teaching

Flipped learning is a teaching method that tries to reverse the traditional teaching process in which teachers mainly assign roles to courses (Aidoo et al., 2022; López-Belmonte et al., 2022). In the past, students were responsibly for reviewing and improving learning in the classroom. Under the flipped teaching method, students first acquire knowledge outside the classroom and then improve it through interactive discussions in the classroom (Long et al., 2017; Verdonck et al., 2022). Most distance teaching courses in Taiwan are involve filming traditional teaching, which then become videos, or teachers directly use “Microsoft Teams” or “Google Meet” to teach, and the teaching method is not significantly different from traditional teaching (Hsiao, 2021). Teachers with ICT competencies and use in flipped learning courses contribute to good practice outcomes of flipped learning (Moreno Guerrero et al., 2021). In addition, teachers introduce flipped learning through gamification courses, which improves students’ learning motivation and learning effectiveness (Mengual-Andrés et al., 2020; Segura-Robles et al., 2020). For students who care about their grades, they can also track group and semester grades through “game-based grouping” to further improve their interest in learning and learning efficiency (Chou et al., 2021). This study aimed to introduce flipped learning and use it in combination with distance online teaching in real-time online distance teaching. It discusses students’ learning attitudes and learning effectiveness to strengthen research on flipped learning in distance online teaching.

Tien et al. (2020) believe that the traditional approaches to teaching hospitality technology courses cause students to only learn via one-way cramming, meaning they are less innovative and lack creative ability as well as a basic understanding of the principles and operating parameters of hospitality technology. Techanamurthy et al. (2020) also believe that hospitality technology courses focus on the teaching of technology and the theory of apprenticeship, while traditional courses do not emphasize the ability to solve problems. Therefore, Chandra et al. (2022) believed that in traditional distance teaching, hospitality technology courses are more difficult to teach through one-way communication, and that they should only be used as supplementary teaching materials. This research aimed to use flipped learning technology to develop individualized acceptance of hospitality technology courses, exploring how they conduct discussions and group activities when gathered together virtually, and how they share and communicate in order to effectively deepen, consolidate, and create hospitality technology, which is the goal of this study.

### Current situation in hospitality courses of distance teaching

In order to solve the problem of ineffective distance teaching on the current hospitality technology courses, and to help students who are slow to learn or have poor basic skills, a new teaching method was introduced in distance teaching. Its purpose is to make hospitality technology courses more adaptable to the characteristics of distance teaching, meaning that can adjust their learning progress according to their basic technical skills and learning ability. In the first step, teachers must provide a complete demonstration operation video of hospitality technology before the distance teaching course for students to learn at home before class. On the day of the main distance course, the content in the teaching video is used for task-based learning interaction, and the learning content is conducted in the form of two-way interactive questioning and discussion, and for the more detailed parts of technical operations, students are invited to ask questions and clarify any points of confusion. In the distance learning classroom, a lot of time is allocated for group discussions. The grouping is based on dividing the worst and best students into the same group according to their past learning results and attitudes, for group discussions and common thinking about innovation and creativity. This approach motivates students in terms of learning attitude and stimulates their interest in learning through flipped learning, effectively improving learning effectiveness, stimulating innovation and creativity, and transforming passive learners into active learners.

The purpose of the present study was to test whether the flipped teaching method can be applied to distance teaching on hospitality technology courses and whether it encourages students to have higher learning effects and better learning attitudes than traditional distance teaching modes. This research is therefore framed around the question: when considering learning effectiveness and learning attitude, is flipped learning distance teaching superior to traditional distance teaching in hospitality technology courses?

## Hypothesis development

### Hospitality technology: The influence of distance online teaching and flipped learning on students’ learning effect

Flipped learning can promote students’ active learning, by making them watch teaching videos before the course and by discussing and solving problems together with the teacher in the course (Bakla, 2018). Flipped teaching can effectively help students participate and gain deeper cognitive knowledge and improve learning effectiveness through more interaction and cooperation among peers (Danker, 2015; Verdonck et al., 2022). The teaching strategy of letting students ask questions through teaching interaction flips the traditional role of learners. With the innovative teaching of forward-looking technology and basic theory that keeps pace with the times, it can not only make the learning process interesting but also optimize the teaching effect (Hung, 2011; Long et al., 2017; López-Belmonte et al., 2022). Flipped learning distance teaching incorporates two-way interaction educational games, thereby stimulating students’ competitive spirit and improving students’ online preparation participation and, therefore, the learning effectiveness for courses (Jo et al., 2018). However, the aforementioned research is based on the research results obtained from flipped learning in traditional brick-and-mortar classroom teaching. Hsiao (2021) believes that not all courses are suitable for remote online teaching. However, Mengual-Andrés et al. (2020) and López-Belmonte et al. (2022) both believe that flipped learning improves students’ learning motivation and learning effectiveness. In the current epidemic situation, the use of distance teaching and the introduction of flipped learning in catering technology courses are the innovation of this research. This study proposes and proves the following hypotheses:

H1: The flipped learning of distance teaching of hospitality technology will affect the learning effect of students.

### Hospitality technology: The influence of distance online teaching and flipped learning on students’ learning attitude

By previewing learning materials before the course, students can effectively save classroom lecture time, reserve more discussion time, and effectively solve their questions. The flipped learning method is actively supported by the students in practice, and compared with the traditional learning method, the students prefer the flipped learning method (Zhang, 2019; Marín Marín et al., 2021). During the COVID-19 epidemic, distance teaching has replaced traditional face-to-face courses. In completely online flipped courses, the high efficiency of online learning and the unique online grouping mechanism has significantly improved learning attitudes (Pyo, 2021). Flipped learning not only significantly improves learning outcomes but also promotes teamwork and innovative and creative thinking among students. It also effectively improves students’ learning attitudes and the interaction between teachers and students (Karabulut-Ilgu et al., 2018; Verdonck et al., 2022).

Basu (2022) believes that flipped learning is a dynamic and interactive learning situation. The teacher is not the protagonist of the course but plays a guiding role, allowing students to apply concepts and creativity and further engage in the learning situation. In the process of flipped learning, students’ learning methods are different from those used in traditional classroom teaching, allowing students to ask more questions and express their thoughts, as well as allowing teachers to further clarify students’ doubts, which has been proved to be effective in medical education in the past (Chen et al., 2017). In the process of changing the learning behavior of students, whether the flipped teaching of distance courses can be the same as the flipped teaching of traditional courses, and whether it has the same effect on learning as traditional courses is worth exploring. Huang and Hsu (2019) believe that although there are many factors affecting learning attitude, students’ learning attitude is mainly composed of three dimensions: cognition, behavior, and affection. Chou et al. (2021) believe that in flipped learning, when students’ learning motivation is enhanced, students are driven to take the initiative to participate in the learning of the course, and then the emotions in the learning attitude will also be driven and positively helpful. Awidi and Paynter (2019) outlined that in the process of flipped learning, students showed interest in learning and strong learning motivation in behavior. Through active participation in the course and active learning before class, they showed specific practical actions. Green (2019) also believes that under flipped learning, students’ understanding of learning subjects is significantly increased, and it has a strengthening effect on the absorption of subject knowledge. Based on the aforementioned research, the present study prosed and proved the following hypotheses:

H2: The flipped learning of distance teaching of hospitality technology will affect students’ learning attitudes.

H2-1: The cognitive dimension of the flipped learning of distance teaching of hospitality technology will affect students’ learning attitude.

H2-2: Flipped learning of distance teaching of hospitality technology will affect the behavioral dimension of students’ learning attitude.

H2-3: The flipped learning of distance teaching of hospitality technology will affect the emotional dimension of students’ learning attitude.

### Hospitality technology: The influence of distance online teaching and flipped learning on students’ learning effectiveness and learning attitude

During the ongoing COVID-19 epidemic, education through distance learning, e-learning, and online media application teaching, has shown a positive correlation between students’ learning effectiveness and learning attitude. If students are not motivated to learn, they cannot achieve good learning outcomes (Putra et al., 2021). The higher the strong learning motivation, the more positive learners learning attitude. It can be seen that learning motivation and learning attitude are also positively related (Awidi and Paynter, 2019). In the field of social and human sciences, learning attitudes were proposed by Rosenberg and Hanland in 1960, and the three elements of attitude are composed of emotion, behavior, and cognition (Mothersbaugh et al., 2020). Therefore, when the learning attitude is induced, the higher the learning attitude, the higher the learning attitude, and the expected goal can be achieved when further learning results are met. This study explores learning effectiveness from the three major elements of learning attitude—cognition, emotion, and behavior, and proposes and proves the following hypotheses:

H3: The learning effect under the flipped learning of the distance teaching of hospitality technology has a positive and significant impact on the cognitive component of learning attitude.

H4: The learning effect under the flipped learning of the distance teaching of hospitality technology has a positive and significant impact on the behavioral tendency of learning attitude.

H5: Learning effectiveness under flipped learning in distance teaching of hospitality technology has a positive and significant impact on the emotional component of learning attitude.

## The establishment of a flipped learning course for distance online teaching

Before the start of the semester, the online teaching platform comprises six units of hospitality technology course content. Before class, students are required to conduct pre-class online learning from the online teaching platform to build the basic concepts of the units. The main purpose of pre-class online learning is to allow students to fully preview the content of the course. Through pre-learning and arousing interest, the students can collect information for in-depth understanding so that they can further innovate and create ideas through self-awareness. To avoid students’ fear of being laughed at or fear of asking teachers questions in the distance online teaching course, the pre-grouping of students was carried out, which will help solve their confusion and help them achieve the ultimate goal of solving the problem through the strength and assistance of their classmates.

In the formal distance online teaching courses for students, teachers used the method of “review” to master students’ learning achievements and innovative ideas, and solve students’ questions and give “step-by-step” technical guidance for technical blind spots. Because long-distance online teaching considers the bandwidth factor in image transmission, most of them are transmitted in SD image quality, which is not so good in terms of image resolution. Therefore, for students’ technical blind spots and technical gaps, during the “camera movement” process, the “shoot a close up” method is adopted, combined with the “slow motion” and “B-roll” skills, so that students can address any problems with learning technology.

Teachers use “game/toy-based e-learning” exercise, such as a creative recipe table (which increases or decreases the ratio of ingredients, and allows new ingredients to be added to create innovative and creative new products), from the process to allow students to share observations, guided by interactive discussions, promotion of collaboration, and creative new product display. Thus, students gain not only the ability to solve problems but also the ability to innovate and create new products.

## Research method

As randomly sampled approaches or assigned research subjects were not suitable for this study, it undertook an effective control according to the existing environment. It adopted a “pre-test and post-test design of different groups in quasi-experimental research” and divided the students into experimental and control groups. The experimental group experienced a situation in which we flipped the learning of distance teaching of hospitality technology, and the control group were taught using traditional learning as part of distance teaching of hospitality technology. The two groups of teaching materials were based on the researcher’s self-edited teaching material—“Traditional learning in distance teaching of hospitality technology”—for 8 weeks of experimental teaching. The impact of “flipped learning of distance teaching of hospitality technology” was explored through students’ learning outcomes and learning attitudes. Both the experimental and control groups received a learning effectiveness test (pre- and post-tests), using two sets of learning attitude questionnaires, and finally, the outcome differences were compared between the two groups.

### Variable measurement of research

In this study, the experimental group who experienced “flipped learning of distance teaching of hospitality technology” and the control group with “traditional learning of distance teaching of hospitality technology” are independent variables. The post-test of “traditional learning in distance teaching of hospitality technology” learning effect and the post-test of “traditional learning in distance teaching of hospitality technology” learning attitude are dependent variables, to reduce experimental interference. Both the groups used the textbook “traditional learning in distance teaching of hospitality technology” compiled by the researchers as the teaching material, which was used as the control variable of this study. Before the “distance teaching of hospitality technology,” the students in the experimental and control groups took the test on “traditional learning in distance teaching of hospitality technology foundation” compiled by the researchers as a pre-test of learning effectiveness. The score of “thematic production questionnaire on learning attitudes of hospitality technology” was used as a pre-test of learning attitude, and the two were used as covariate variables in this study. Experimental group and Control group students in “distance teaching of hospitality technology”, using the “traditional learning in distance teaching of hospitality technology study” compiled by the researchers as the post-test of learning effectiveness. The score of the “thematic production questionnaire on learning attitudes of hospitality technology” was used as a post-test of learning attitude, and the two were used as dependent variables in this study.

### Learning attitude questionnaire

Learning attitudes include cognitive components, affective components, and behavioral tendencies (Mothersbaugh et al., 2020). This study builds on previous work by Mothersbaugh et al. (2020), including the “thematic production-questionnaire on learning attitudes of hospitality technology” using a Likert seven-component table, with “1” strongly disagreed and “7” strongly agreed.

### Research objects and sampling data

In this study, through experiments, 55 college students in the third grade of the department of hospitality management of a university in Taiwan completed an 8-week flipped learning course (4 h per week, 32 h in total). Questionnaires were distributed using a questionnaire survey method. In this study, 55 questionnaires were collected, 53 valid questionnaires were taken into account, and the effective recovery rate was 96.4%. In terms of gender ratio, male students accounted for 45.3% and female students accounted for 54.7%. In terms of admission channels, ordinary high schools accounted for 13.2%, higher vocational schools accounted for 69.7%, and comprehensive high schools accounted for 17%.

### Statistical tools

Questionnaire data were analyzed using SPSS software. The reliability and validity of the recovered questionnaires were analyzed, and the variance analysis was used to explore the differences in students’ learning effectiveness and learning attitudes under the flip learning of distance teaching of hospitality technology. Finally, regression analysis was used to explore the relationship between learning effectiveness and learning attitude and to verify the research hypotheses.

### Validity and reliability analysis

#### Expert validity

The expert validity is content validity in this research. Experts and scholars in related fields of education, including two professors in the field of education and two practical experts in the field of hospitality and education services, were hired to assist in reviewing the content and format of this questionnaire, the appropriateness of the topic, completeness, layout, etc., and to give comments and review and amend. This study was revised based on the opinions provided by the experts and scholars to confirm the expert validity of this questionnaire.

#### Reliability analysis

This research scale uses Cronbach’s α as the internal consistency reliability analysis of this scale. DeVellis (1991) believes that the internal consistency reliability coefficient of the subscale should be accepted above 0.70. Therefore, this research tool has been verified by reliability analysis, and the reliability of the subscales is between 0.77 and 0.83. The cognitive component of learning attitude was 0.78, the affective component was 0.79, and the behavioral tendency was 0.83. It shows that this questionnaire has good internal consistency reliability in learning attitude. The final formal questionnaire consists of 15 items, and the questionnaire in this study has a high reliability.

## Data analysis and proving hypotheses

This study explores the effect of “flipped learning of distance teaching of hospitality technology” on learning outcomes and attitudes toward learning. “Learning” is an independent variable, with “traditional learning in distance teaching of hospitality technology” learning effect post-test and “traditional learning in distance teaching of hospitality technology” learning attitude post-test as a dependent variable, “subject” as a control variable for analyzing the learning effect and learning attitude of “traditional learning in distance teaching of hospitality technology.”

### “Flipped learning of distance teaching of hospitality technology” on the difference analysis of students’ learning effect

Learning outcomes refer to students’ responses and changes in their learning behavior after participating in distance learning activities (Guay et al., 2008; Pike et al., 2012). This study explores whether students in the flipped learning group and the traditional learning group have significant differences in the learning outcomes of traditional learning in distance teaching of hospitality technology after undergoing the “flip learning of distance teaching of hospitality technology” experiment.

#### “Flipped learning of distance teaching of hospitality technology” pre-test analysis of learning effectiveness

In the pre-test based on “traditional learning in distance teaching of hospitality technology” between the experimental and control groups, the average score of the experimental group was 47.08, with a standard deviation of 11.89, and the average score of the control group was 48.81, with a standard deviation of 14.01. The *t*-value was −0.486, and *P* = 0.327 did not reach a significant level of 0.05. Therefore, before the experiment, the two groups of students had similar learning outcomes based on “traditional learning in distance teaching of hospitality technology,” and there was no significant difference between the two groups, as shown in Tables 1, 2.

**TABLE 1:** The standard deviation of pretest results for learning effectiveness.

	Experimental group (*N* = 26)		Control group (*N* = 27)		*T*-value
	Average	Standard deviation	Average	Standard deviation	
Pre-test score	47.08	11.89	48.81	14.01	−0.486

*P* > 0.05.

**TABLE 2:** The statistical analysis of pre-test results of learning effectiveness.

SV	SS	df	MS	*F*	*P*
Group	51.046	1	51.046	0.773	0.619
Pre-test score	11.137	1	11.137	0.169	
Group * pre-test score	16.566	1	16.566	0.254	
Error	3234.491	49	66.010		

#### “Flipped learning of distance teaching of hospitality technology” post-test analysis of learning effectiveness

According to the teaching scope of “Traditional learning in distance teaching of hospitality technology,” post-test questions were prepared. The post-test effect of “flip learning of distance teaching of hospitality technology” was tested, and the post-test effect of “traditional learning in distance teaching of hospitality technology” on “flip learning of distance teaching of hospitality technology” was analyzed by single-factor covariate analysis. Before conducting the ANCOVA, the basic assumption of the same value of the regression coefficients should be met, so the homogeneity test of the regression coefficients within the group should be carried out first. The results of the intragroup regression coefficient homogeneity test are shown in Table 2. The interaction effect of “group × pre-test” did not reach a significant level, indicating that there was no significant difference in the slope formed by the two groups before and after the test, which was consistent with the homogeneity of the regression coefficient within the group [*F*_(1,49)_ = 0.254, *p* = 0.619 > 0.05]. ANCOVA can be continued if it meets the specification.

Covariate (ANCOVA) was used for statistical analysis. The mean and standard deviation of the pre-test and post-test scores of the experimental and control groups are shown in Table 3, and the summary table of the ANCOVA is shown in Table 4.

**TABLE 3:** The ANCOVA analysis of pre-test and post-test results of learning effectiveness.

Group	People	Pre-test score		Post-test score		Adjusted	
		Average	Standard deviation	Average	Standard deviation	Average	Standard deviation
EG	26	47.08	11.89	77.15	8.767	77.179	1.583
CG	27	48.81	14.01	65.22	7.170	65.198	1.554

**TABLE 4:** The significant results of ANCOVA analysis of pre-test and post-test results of learning effectiveness.

Source	SS	df	MS	*F*	Significance
Pre-test score	6.995	1	6.995	0.108	
Group	1892.546	1	189.546	29.107	0.000
Error	3251.057	50	65.021		
Total	272885.000	53			

### “Flipped learning of distance teaching of hospitality technology” difference analysis of students’ learning attitude

This study explores whether students in the flipped learning group and the traditional learning group have significant differences in their learning attitudes after undergoing the “flip learning of distance teaching of hospitality technology” experiment.

#### “Flipped learning of distance teaching of hospitality technology” pre-test analysis of students’ learning attitude

The experimental and control groups had an average score of 3.623, with a standard deviation of 0.347, in the pre-test of “traditional learning in distance teaching of hospitality technology”; the average score of the control group was 3.301, with a standard deviation of 0.396. The *t*-value was 2.851, and *P* = 0.461 did not reach a significant level of 0.05. Therefore, before the experiment, the two groups of students had similar learning attitudes based on “traditional learning in distance teaching of hospitality technology,” and there was no significant difference between the two groups, as shown in Tables 5, 6.

**TABLE 5:** The standard deviation of pretest results for learning attitude.

	Experimental group (*N* = 26)		Control group (*N* = 27)		*T*-value
	Average	Standard deviation	Average	Standard deviation	
Pre-test score	3.623	0.347	3.301	0.396	2.851

*P* > 0.05.

**TABLE 6:** The statistical analysis of pre-test results of learning attitude.

SV	SS	df	MS	*F*	*P*
Group	0.014	1	0.014	0.115	0.736
Pre-test score	0.016	1	0.016	0.137	
Group * pre-test score	0.092	1	0.092	0.775	
Error	5.802	49	0.118		

#### “Flipped learning of distance teaching of hospitality technology” post-test analysis of students’ learning attitude

The experimental and control groups took a post-test analysis of learning attitudes on “traditional learning in distance teaching of hospitality technology,” with single-factor covariates to analyze the post-test effect. Before conducting the ANCOVA, the basic assumption of the same value of the regression coefficients should be met, so the homogeneity test of the regression coefficients within the group should be carried out first. The results of the intragroup regression coefficient homogeneity test are shown in Table 6. The interaction effect of “group × pre-test” did not reach a significant level, indicating that there was no significant difference in the slope formed by the two groups before and after the test, which was consistent with the homogeneity of the regression coefficient within the group [*F*_(1,49)_ = 0.775, *p* = 0.383 > 0.05], ANCOVA can be continued if it meets the specification.

The mean and standard deviation of the pre-test and post-test scores of the experimental and control groups are shown in Table 7, and the summary table of ANCOVA is shown in Table 8.

**TABLE 7:** The ANCOVA analysis of pre-test and post-test results of learning attitude.

Group	People	Pre-test score		Post-test score		Adjusted	
		Average	Standard deviation	Average	Standard deviation	Average	Standard deviation
EG	26	3.623	0.347	4.980	0.337	4.975	0.070
CG	27	3.301	0.396	3.854	0.343	3.859	0.069

**TABLE 8:** The significant results of ANCOVA analysis of pre-test and post-test results of learning attitude.

Source	SS	df	MS	*F*	Significance
Pre-test score	0.007	1	0.007	0.058	
Group	14.231	1	14.231	120.734	0.000
Error	2.894	50	0.118		
Total	1051.684	53			

### “Flipped learning of distance teaching of hospitality technology” difference analysis of students’ learning attitude

In this study, after 8 weeks of teaching experiments, the learning attitude of the students in the experimental and control groups of “flipped learning of distance teaching of hospitality technology” were explored. Independent one-way covariate analysis was carried out taking experimental group “flip learning at distance teaching of hospitality technology” and control group “traditional learning at distance teaching of hospitality technology” as independent variables, and the pre-and post-test scores of the two groups of students as covariate variables, and the later test scores as disguised. In the experimental and control groups, an analysis and discussion on the “traditional learning in distance teaching of hospitality technology” learning attitude questionnaire was conducted, and the scores of three dimensions (cognition, behavior, and emotion) were analyzed.

#### Pre-test analysis of “traditional learning in distance teaching of hospitality technology” learning attitude questionnaire

The pre-test of the “traditional learning in distance teaching of hospitality technology” learning attitude questionnaire was conducted before the implementation of teaching activities. The valid samples included 26 students in the experimental group and 27 students in the control group, with a total of 53 people. The descriptive statistical results of the pre-test of the “traditional learning in distance teaching of hospitality technology” learning attitude questionnaire of the two groups are shown in Table 9.

**TABLE 9:** The statistical analysis of pre-test results in different dimensions of learning attitude.

Dimension	Group	Number of people	Average	Standard deviation	*T*-value
Cognition	Experimental group	26	3.523	0.585	1.818
	Control group	27	3.237	0.560	
Behavior	Experimental group	26	3.646	0.675	2.140
	Control group	27	3.244	0.691	
Affection	Experimental group	26	3.700	0.607	1.018
	Control group	27	3.511	0.735	

In the “cognition” dimension, the average value of the students in the experimental group was 3.523, the standard deviation was 0.585, the average value of the students in the control group was 3.237, the standard deviation was 0.560, and the *t*-value was 1.818 (*P* > 0.05). In the “behavior” dimension, the average value of the students in the experimental group was 3.646, the standard deviation was 0.675, the average value of the students in the control group was 3.244, the average difference was 0.691, and the *t*-value was 2.140 (*P* > 0.05). The value was 3.700, the standard deviation was 0.607, the student means of the control group was 3.511, the standard deviation was 0.735, and the *t*-value was 1.018 (*P* > 0.05). There is no significant level in the mean values measured before the experimental and control group in the above three dimensions (cognition, behavior, and emotion), and there is no significant difference in the learning attitude of the two groups of students before the experimental teaching.

#### Post-test analysis of “traditional learning in distance teaching of hospitality technology” learning attitude questionnaire

We further explored whether “flipped learning of distance teaching of hospitality technology” and “traditional learning in distance teaching of hospitality technology” have a significant effect on the learning attitude of “thematic production–hospitality management technology” of the two groups of students. An independent sample of one-way covariate analysis was performed. In this study, an independent sample single-factor covariates analysis is carried out, with the pre-test scores of the “traditional learning in distance teaching of hospitality technology” learning attitude questionnaire of the two groups of students used as covariate variables; the teaching of “flip learning of distance teaching of hospitality technology” and “traditional learning in distance teaching of hospitality technology” methods as independent variables; and the “traditional learning in distance teaching of hospitality technology” learning attitude questionnaire as a dependent variable. After excluding the interference of the pre-test scores, the “thematic production–hospitality management technology” learning attitude, it was determined whether there is a significant difference in the statistical analysis.

#### Descriptive statistics

Before conducting an independent sample single-factor covariate analysis, first, descriptive statistics for the problems of each degree was performed, and the differences between the experimental and control groups were examined. The descriptive statistics are shown in Table 10.

**TABLE 10:** The statistical analysis of post-test results in different dimensions of learning attitude.

Dimension	Question	Experimental group		Control group	
		Average	Standard deviation	Average	Standard deviation
Cognition	C01. I think learning hospitality technology can increase my chances of connecting with the world’s hospitality culture.	4.65	1.468	3.19	1.861
	C02. I think that learning hospitality technology will help me develop the strength for further education and employment in the future.	4.73	1.687	3.59	1.500
	C03. I think learning hospitality technology can help me have better employment opportunities.	5.38	1.098	4.15	1.512
	C04. I think that learning hospitality technology helps absorb international hospitality culture.	4.65	1.495	3.70	1.382
	C05. I think learning hospitality technology can expand my knowledge.	5.00	1.131	4.37	1.391
Behavior	B01. As long as I have questions in the study of hospitality technology, I will try my best to solve them, such as discussing with classmates, looking up books, and asking teachers for advice.	4.50	1.421	3.89	1.601
	B02. I will take the initiative to answer the questions asked by the teacher when I take the hospitality technology course.	5.27	1.282	3.67	1.797
	B03. Every time I finish a new hospitality technology course, I will take the initiative to review.	5.08	1.383	4.26	1.655
	B04. I will often participate in activities related to hospitality technology courses.	5.08	1.598	3.63	1.621
	B05. Even without the supervision of the teacher, I will still do my homework or activity business related to hospitality technology.	4.88	1.505	4.48	1.528
Affection	E01. I think it is a pleasure to take the hospitality technology course.	4.77	1.773	3.74	1.403
	E02. I am very concerned about my grades in the hospitality technology course.	5.62	1.444	3.56	1.908
	E03. Learning hospitality technology gives me a sense of accomplishment.	4.42	1.362	3.93	1.730
	E04. I like to discuss with my classmates hospitality technology courses.	5.23	1.657	3.37	1.822
	E05. I like to learn about hospitality technology.	5.08	1.647	4.15	1.406

#### Covariate variance analysis of regression coefficient homogeneity test

Before the covariate analysis, the regression coefficient homogeneity test is required. The homogeneity test results of the two groups of students’ “traditional learning in distance teaching of hospitality technology” learning attitude before and after the test are shown in Table 11.

**TABLE 11:** Homogeneity test of regression coefficients of post-test results of different dimensions of learning attitude.

Dimension	SV	SS	df	MS	*F*	*P*
Cognition	Group	0.000	1	0.000	0.001	0.976
	Pre-test score	0.015	1	0.015	0.040	0.843
	Group * pre-test score	0.431	1	0.431	1.176	0.283
	Error	17.951	49	0.366		
Behavior	Group	1.569	1	1.569	3.390	0.072
	Pre-test score	1.297	1	1.297	2.803	0.100
	Group * pre-test score	0.431	1	0.431	0.932	0.339
	Error	22.678	49	0.463		
Affection	Group	0.034	1	0.034	0.060	0.807
	Pre-test score	0.076	1	0.076	0.136	0.714
	Group * pre-test score	0.428	1	0.428	0.768	0.385
	Error	27.309	49	0.557		

The results of the homogeneity test of the regression coefficient within the group show that the *F*-value statistic did not reach a significant level (cognition: *F* = 1.176, *p* = 0.283 > 0.05; behavior: *F* = 0.932, *p* = 0.339 > 0.05; emotion: *F* = 0.768, *p* = 0.385 > 0.05), so there is no difference between the covariate variables (pre-test score of learning attitude) and the dependent variable (post-test score of learning attitude) because of the different processing levels of the independent variable, which is consistent with the regression within the covariate array. Covariate analysis can be continued with the assumption of coefficient homogeneity.

#### One-way covariate analysis

The pre-test scores of the “traditional learning in distance teaching of hospitality technology” learning attitude questionnaire of the two groups of students were used as covariates, with the teaching method as an independent variable, and the post-test scores as variables for single-factor covariate analysis. The mean, standard deviation, and adjusted mean of the “traditional learning in distance teaching of hospitality technology” learning attitude questionnaire are shown in Tables 12, 13.

**TABLE 12:** The ANCOVA analysis of pre-test and post-test results of different dimensions of learning attitude.

Dimension	Group	People	Pre-test score		Post-test score		Adjusted	
			Average	Standard deviation	Average	Standard deviation	Average	Standard deviation
Cognition	EG	26	3.523	0.585	4.954	0.488	4.926	0.122
	CG	27	3.237	0.560	3.830	0.692	3.811	0.120
Behavior	EG	26	3.646	0.675	4.962	0.577	4.941	0.140
	CG	27	3.244	0.691	3.985	0.788	4.058	0.136
Affection	EG	26	3.700	0.607	5.023	0.749	5.015	0.148
	CG	27	3.511	0.735	3.748	0.731	3.730	0.145

**TABLE 13:** The significant results of ANCOVA analysis of pre-test and post-test results of different dimensions of learning attitude.

Dimension	SV	SS	df	MS	*F*	*P*
Cognition	Pre-test score	0.019	1	0.019	0.051	0.823
	Group	15.456	1	15.456	42.050	0.000
	Error	18.382	50	0.368		
	Total	1052.440	53			
Behavior	Pre-test score	1.366	1	1.366	2.956	0.092
	Group	9.415	1	9.415	20.370	0.000
	Error	23.109	50	0.462		
	Total	1093.320	53			
Affection	Pre-test score	0.176	1	0.176	0.318	0.575
	Group	21.649	1	21.649	39.024	0.000
	Error	27.737	50	0.555		
	Total	1063.240	53			

## Discussion

### Difference analysis of the learning effect of “flip learning of distance teaching of hospitality technology”

The statistical results show that the learning effect of “traditional learning in distance teaching of hospitality technology” after the post-test adjustment in the experimental group is *M* = 77.179. In the control group is *M* = 65.198 (the adjusted average difference between the experimental group and the control group is statistically significant level *F*_(1,50)_ = 29.107, *p* = 0.000 < 0.05), and the result reached a significant level, which means that after controlling the conditions of the pre-test and excluding the influence of the pre-test score, in the two groups in the “traditional learning in distance teaching” of hospitality technology, there were significant differences in post-test results consistent with the hypothesis. López-Belmonte et al. (2022) proposed that flipped learning can significantly improve students’ learning outcomes. Moreno Guerrero et al. (2021) also mentioned that flipped learning has good practical ability in teaching. Therefore, this research introduces hospitality technology in distance online teaching into flipped learning, which is fully feasible in practical operation, consistent with theoretical verification, and echoes the arguments of the aforementioned scholars. This is consistent with hypothesis H1: Flip learning of distance teaching of hospitality technology will affect students’ learning outcomes.

### Analysis of differences in learning attitudes towards “flipped learning of distance teaching of hospitality technology”

The statistical results show that the learning attitude of “Traditional learning in distance teaching of hospitality technology” after the post-test adjustment in the experimental group is *M* = 4.975, and that in the control group is *M* = 3.859 [the adjusted average difference between the experimental and control groups is statistically significant, with *F*_(1,50)_ = 120.734, *p* = 0.000 < 0.05], and the result reached a significant level, which means that after controlling the conditions of the pre-test and excluding the influence of the pre-test score, the two groups in the “traditional learning in distance teaching,” there were significant differences in the post-test scores of learning attitudes of hospitality technology. Mengual-Andrés et al. (2020) proposed that flipped learning will generate greater motivation for students, resulting in significant improvements in autonomy, motivation, and self-esteem. López-Belmonte et al. (2022) also believed that flipped learning significantly improved students’ learning attitudes in terms of autonomy, food and sex, interaction, and enthusiasm. This research has been verified by practice and echoes the academic arguments of the aforementioned scholars. Hospitality technology distance online teaching introduces flipped learning, which can effectively improve students’ learning attitude in practice. This is consistent with hypothesis H2: Flip learning of distance teaching of hospitality technology will affect students’ learning attitudes.

The results of the single-factor covariate analysis of this study showed that excluding the influence of the pre-test scores of learning attitudes, the adjusted mean of the “cognition” post-test was *M* = 4.926 for the experimental group and *M* = 3.811 for the control group (*F* = 42.050, *p* = 0.000 < 0.05), consistent with the hypothesis H2-1: Flip learning of distance teaching of hospitality technology has a positive and significant impact on the cognitive component of learning attitude is established. The adjusted mean of the “behavior” post-test was *M* = 4.941 for the experimental group and *M* = 4.058 for the control group (*F* = 20.370, *p* = 0.000 < 0.05), indicating that hypothesis H2-2 on flipped learning of distance teaching of hospitality technology is correct. The hypothesis that the behavioral tendency of learning attitude has a positive and significant effect is established. In the “emotion” post-test, the adjusted mean of the experimental group is *M* = 5.015 and that of the control group is *M* = 3.730 (*F* = 39.024, *p* = 0.000 < 0.05), which shows that the H2-3 flip learning of distance teaching of hospitality technology is correct. The hypothesis that the affective component of learning attitude has a positive and significant effect is established. Chou et al. (2021) believe that flipped learning has a positive impact on cognition, affection, and behavior of learning attitudes. In this study, flipped learning was applied to long-distance online teaching of hospitality technology, and the same theoretical verification was obtained by Chou et al. (2021).

### “Flipped learning of distance teaching of hospitality technology”: The relationship between learning effectiveness and learning attitude dimension

#### The relationship between learning effectiveness and the cognitive dimensions of learning attitude

As can be seen from Table 14, the results of regression analysis show that the learning effect (β = 0.331) under flip learning of distance teaching of hospitality technology has a significant impact on the cognitive component of learning attitude, so H3 is established.

**TABLE 14:** The relationship between learning effectiveness and cognitive dimensions of learning attitude.

Learning effectiveness (independent variable)	Learning attitude (dependent variable)					
	Cognition component		Behavior component		Affection component	
	β	*p*	β	*p*	β	*p*
Learning effectiveness	0.331	0.015	0.251	0.070	0.386	0.004
*F*	6.290		3.422		8.913	
Significance	0.015		0.070		0.004	
*R* ^2^	0.110		0.063		0.149	
Adjusted *R*^2^	0.092		0.045		0.132	

#### The relationship between learning effectiveness and learning attitude and behavior dimension

According to Table 14, the results of regression analysis show that the learning effect (β = 0.251) under flip learning of distance teaching of hospitality technology has a slightly significant impact on the behavioral component of learning attitude (0.10 < *P* < 0.05), so H4 is established.

#### The relationship between learning effectiveness and learning attitude and dimension

Table 14 indicates that the results of the regression analysis show that the learning effect (β = 0.386) under flip learning of distance teaching of hospitality technology has a significant impact on the emotional component of learning attitude; hence, H5 is established. In this study, there was no significant effect between learning effectiveness, cognition, affection, and behavior of learning attitude, which is different from the results of another previous study by Chou et al. (2021). In this study, the relationship between learning effectiveness and cognition, affection, and behavior of learning attitudes under the hospitality technology of distance online teaching flipped learning can be used by hospitality departments as a reference when designing future distance online teaching.

## Conclusions

Based on the research results of the aforementioned data analysis, the present study analyzed the influence of the teaching method of “flipped learning of distance teaching of hospitality technology” on the learning effect and learning attitude of students.

### Analysis of the impact of “flipped learning of distance teaching of hospitality technology” on students’ learning effects

In this study, under the condition that the experimental group and the control group had no significant influence on the pre-test learning effectiveness, the students in the experimental group scored higher than the students in the control group, and there was a significant difference in the post-test results of the learning effect. Experimental group students believe that “flip learning of distance teaching of hospitality technology” is of substantial help for them to learn food and travel technology. Pre-class online video teaching and pre-class exercises enable students to be self-aware and understand what they do not understand, which can be used in online teaching classes. Through question discussion and teacher demonstration answers, it helps to achieve learning results. Students in the control group can only watch teaching videos online in real time. Apart from the lack of two-way communication opportunities between teachers and students, students in control group could not alleviate uncertainties through questions, discussions, or individual teaching. The learning effect in the control group is lower than that of the students in the experimental group. In this study, the results of the “flip learning of distance teaching of hospitality technology” teaching method on students’ learning effectiveness echo the research results of Bakla (2018), Zhang (2019), Pyo (2021), and López-Belmonte et al. (2022). The teaching method of “hospitality technology” is significantly more effective for the students of the experimental group than the students of the control group. Therefore, the introduction of flipped learning into “hospitality technology” distance online teaching is helpful for students’ learning effectiveness, and the research results can be used a reference for hospitality-related departments in the future.

### Analysis of the influence of “flip learning of distance teaching of hospitality technology” on students’ learning attitude

In this study, excluding the influence of the pre-test scores of learning attitudes, students achieved significant differences in the dimensions of “cognition,” “behavior,” and “emotion” in learning attitudes. The students of the experimental group in the “flip learning of distance teaching of hospitality technology” teaching method are conducted in groups during distance teaching. Therefore, students with poor learning results can adjust their self-learning methods under the guidance, assistance, and drive of their peers, enabling their learning to keep pace with the group, showing a more positive attitude in the three areas of cognition, behavior, and emotion. Chou et al. (2021) and Moreno Guerrero et al. (2021) put forward the argument that flipped teaching has a positive and significant impact on students’ learning attitudes. On the other hand, for control group students who experienced the “traditional teaching of distance teaching of hospitality technology,” the students were independent individuals at the moment of traditional distance online teaching. The formats of traditional distance online teaching courses involve a tutor leading the course in one direction. There is no real-time interactive discussion among peers, resulting in almost no interaction among classmates. When students suffer from incomprehension, doubts, frustrations, etc., while the course is in progress, teachers cannot immediately solve students’ confusion, and the students can only ask teachers for help after class. When you are afraid, you may give up solving puzzles or even give up the course. In this situation, without the drive and promotion of peers, it is easy to show the situation of giving up, escaping, or even refusing to study in the attitude of course learning. Therefore, the results of the study echo the results of Karabulut-Ilgu et al. (2018). López-Belmonte et al. (2022) found that in the process of flipped teaching, teamwork and innovative thinking among students can help improve students’ learning attitudes. Awidi and Paynter (2019) and Chou et al. (2021) proposed that when students are motivated to learn, it will help them improve their learning attitude and teaching effectiveness. The teaching method of “flip learning of distance teaching of hospitality technology” in this study is the most important in terms of cognition. There are also differences in teaching flipped learning on physical and online courses due to the students’ learning attitudes. Therefore, “hospitality technology” distance online teaching introduces flipped learning, which is helpful for students’ learning attitude. In terms of the importance of learning attitude, the results of the study are cognition, affection, and behavior, and the findings of this study can be used as a reference for the distance online teaching of hospitality-related departments.

### Analysis of the relationship between the learning effect and the learning attitude dimension of “flipped learning of distance teaching of hospitality technology”

This study explores the relationship between the learning effect and the learning attitude dimension of “flipped learning of distance teaching of hospitality technology” using regression analysis. The results showed that there were no significant correlations between the cognitive and emotional dimensions of learning outcomes and learning attitudes. The behavioral dimension is only slightly significantly correlated. The main reason for exploring this is whether students can actively study, review, and participate. If the students still have passive learning behavior attitudes, then they can improve their willingness to learn through flipped learning and classmate support. The main problem is that when the support force disappears, learning behavior may return to the original passive state.

### Impacts and research limitations of this research

This study draws the conclusion that flipped learning in long-distance online teaching of hospitality technology helps to improve students’ learning effectiveness and learning attitude, serving as a stepping stone for hospitality technology-related departments of long-distance online teaching and flipped learning. This research method responds to the needs of today’s new generation of students and is validated in practice by this study. After the research is completed, the effectiveness of long-distance online teaching of hospitality technology in hospitality-related departments can be further strengthened to reduce the impact of COVID-19. As shown by the statistically significant results of this study, flipped learning can indeed strengthen traditional distance online teaching of hospitality technology. Similarly, after this study, a new generation of hospitality technology distance online teaching foundation for hospitality-related departments can be established, and the use of flipped learning methods can provide an effective teaching and learning experience for students, with learning effects closer to traditional physical courses.

Therefore, this research will have a series of relevant impacts on the distance online teaching of hospitality technology in future hospitality-related courses, which are included as follows:

1. Update the traditional hospitality technology distance online teaching methods and means, with learners as the main body and teachers as the auxiliary role, to impart professional skills.

2. Through the grouping and game-like methods of flipped learning, the situation of students without peer interaction during the traditional hospitality technology distance online teaching can be improved. Through the power of peers, students can strengthen learning effectiveness and learning attitude, and successfully seek answers to schoolwork confusion.
3. The traditional hospitality technology long-distance online teaching fixed a single lens for teaching obstacles. Through the flipped learning method of this study, the use of pre-class learning, class review, and real-time discussion during the course can fully meet students’ problems and blind spots.

The main limitation of this research was the investigation object because most of the students of the university of science and technology were graduates of higher vocational colleges, and they are practical technical students. Therefore, there may have been a lack of academic and theoretical students who have graduated from ordinary high schools or universities. Therefore, as a future research direction, we will seek to expand the research sample to different cities with common universities and high similarity with this research. In this way, not only can we compare the differences between the goals of ordinary universities and universities of science and technology, whilst also furthering research on the cultural differences in different cities, and the impact of flipped learning on students’ learning effectiveness, and learning attitudes.

## Data availability statement

The original contributions presented in this study are included in the article/supplementary material, further inquiries can be directed to the corresponding author.

## Ethics statement

The present study was conducted in accordance with the recommendations of Ming Chuan University and Tajen University, with written informed consent obtained from all the participants. All the participants were asked to read and approve the ethical consent form before participating in the present study. The participants were also asked to follow the research guidelines as mentioned in the consent form.

## Author contributions

K-WC contributed to conception, design of the study, and wrote sections of the manuscript. Z-YW wrote the first draft of the manuscript. Both authors contributed to manuscript revision, read, and approved the submitted version.
